# Elastography Evaluation of Benign Thyroid Nodules in Patients Affected by Hashimoto's Thyroiditis

**DOI:** 10.1155/2015/367054

**Published:** 2015-07-27

**Authors:** Carlo Cappelli, Ilenia Pirola, Elena Gandossi, Annamaria Formenti, Barbara Agosti, Maurizio Castellano

**Affiliations:** Department of Clinical and Experimental Sciences, Endocrine and Metabolic Unit, University of Brescia, Piazzale Spedali Civili No. 1, 25100 Brescia, Italy

## Abstract

The aim of the present prospective study was to evaluate the predictive value of elastography in benign thyroid nodules of patients affected by Hashimoto's thyroiditis (HT). From January 2011 to January 2012, 242 nodules in patients affected by HT were submitted to fine needle aspiration cytology (FNAC). All of the patients underwent sonography and elastography performed before FNAC. 230 (95%) nodules were benign, 8 papillary cancers, and 4 follicular lesions. Score 1 was found in 79.1% of benign lesions (sensitivity 79.1%; specificity 66.7%; PPV 97.8%; NPV 14.3%; accuracy 78.5%; *p* < 0.05). In order to evaluate the outcome of thyroid ultrasound echogenicity in relation to elastography features of nodule(s), all the patients with benign nodules were stratified according to their hypoechoic pattern of thyroid (mild-moderate and severe). Following stratification score 1 was found in 84.2% of benign nodules (sensitivity 75.0%; specificity 88.9%; PPV 27.3%; NPV 98.4%; accuracy 88.2%; *p* < 0.0001) of patients with a mild-moderate ultrasound thyroid hypoechogenicity, whereas it was found in 60% of benign nodules (*p* = 0.715) of patients with a marked thyroid hypoechogenicity. Elastography appears to have limited value in detecting thyroid cancer in patients affected by Hashimoto's thyroiditis with severe hypoechoic thyroid tissue.

## 1. Introduction

Hashimoto's thyroiditis (HT), also known as chronic lymphocytic thyroiditis, is an autoimmune disease characterized by lymphocytic infiltration of the gland and production of autoantibodies that target thyroid peroxidase and/or thyroglobulin, resulting in tissue destruction and progressive loss of thyroid function [1].

After ultrasound (US) introduction into clinical practice in the late 1960s, thyroid ultrasonography proved to be very effective in the diagnostic approach to thyroid disorders [2–4]. Many studies have investigated whether the ultrasonographic characteristics of thyroid nodules are useful indicators of histological malignancy.

Overall, these investigations suggest a few ultrasonographic features that are significantly more frequent in malignant than in benign thyroid nodules, and some have tried to define a set of characteristics that identify nodules at higher risk of malignancy [5].

The characteristic US pattern of Hashimoto's glands consists of an array of tiny hypoechoic nodules that may become confluent, interspersed with echogenic fibrous bands [6]; nevertheless, US should be interpreted with caution due to the diffuse heterogeneity and the presence of pseudonodules related to ongoing inflammation. Elastography is a method which assesses the risk of the malignancy and provides information about the degree of hardness in tissue. Recently elastography has been proposed as a new technique for differentiating pseudonodules from nodules in HT [7] and benign from malignant lesions of thyroid gland [5, 8–10]. HT is able to change the hardness of the thyroid tissue. There have been few studies that have shown using elastography that HT is able to change the hardness of the thyroid gland decreasing its capacity to distinguish benign from malignant nodule [11, 12].

The aim of the present prospective study was to evaluate the predictive value of elastography in the characterization of benign thyroid nodules of patients affected by HT.

## 2. Materials and Methods

From January 2011 to January 2012, 250 consecutive patients affected by HT with at least one solid nodule were submitted to fine needle aspiration cytology (FNAC). Cases were selected from patients referred to our thyroid unit for US-guided FNAC. The selection criteria were as follows: (1) indication to FNAC in accordance with the major guidelines [5, 14]; (2) positive test for thyroglobulin antibody (TgAb) and/or thyroid peroxidase antibody (TPOAb) and a thyroid gland with hypoechoic pattern at US evaluation.

All of the patients underwent sonography and elastography performed by the same skilled sonographer (CC) before US-guided FNAC. Thyroid sonography and elastography were performed with a real-time instrument (Vision 900; Hitachi Medical System, Tokyo, Japan) equipped with a linear probe with a central frequency of 6–13 MHz.

**Figure 1:**
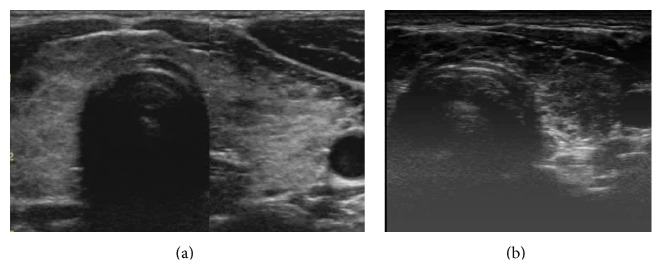
(a) Hashimoto's thyroiditis with mild sonographic hypoechogenicity. (b) Hashimoto's thyroiditis with severe sonographic hypoechogenicity.

The sonographic hypoechogenicity of thyroid gland (Figures 1(a) and 1(b)) was classified from mild-moderate to severe in accordance with literature [15–18].

Findings at elastography were classified according to the elasticity scores by Rago et al. [13]. Briefly, score 1 indicated nodules with high elasticity, score 2 nodules with intermediate elasticity, and score 3 nodules with low elasticity (Table 1). To minimize the intraobserver variability, the freehand compression applied on the neck was standardized by real-time measurement displayed on a numeric scale (graded 1–5) to maintain an intermediated level optimal for elastographic evaluation [6, 8].

The serum concentrations of TgAb (normal range: <60 U/mL) and TPOAb (normal range: <60 U/mL) were measured using immunochemiluminescent assays employing commercial kits (Brahms, Hennigsdorf, Germany).

All patients gave their informed consent to participate in the study, which was performed in accordance with the Declaration of Helsinki.

All data were analyzed with SPSS version 17 software (SPSS Inc., Chicago, IL). Comparisons between groups were performed by an analysis of variance general linear model; a *χ* ^2^ test was used for categorical variables. Statistical significance was considered at *p* < 0.05.

## 3. Results

Two hundred fifty consecutive patients (202 female, 48 male), mean age 52.2 ± 11.2 years, were submitted to US-guided FNAC.

Of the 262 nodules submitted to cytology, 20 (7.6%) appeared to be nondiagnostic, and as a consequence 16 patients were excluded from the study. Among 234 patients two hundred forty-two nodules were collected: 230 (95%) were benign, 8 papillary cancers, and 4 follicular lesions. Histology confirmed 8 papillary and 4 follicular cancers.

The lesions classified as scores 1, 2, and 3 on elastography are listed in Table 2.

Score 1 was found in 182/230 (79.1%) of benign lesions (sensitivity 79.1%; specificity 66.7%; positive predictive value 97.8%; negative predictive value 14.3%; accuracy 78.5%; *p* < 0.05).

All 227 patients with benign nodules were stratified in accordance with the echogenic pattern of thyroid gland (mild-moderate/severe): 128 (56%) patients presented a mild-moderate thyroid echogenicity (Group A) and 99 (44%) a severe one (Group B). The two groups were then stratified in accordance with the elastographic scoring of the nodule (scores 1, 2, and 3). The two groups were superimposable for gender (86/42 versus 64/35 F/M), age (53.1 ± 12.8 versus 49.6 ± 10, years), and TSH values (3.9 ± 1.1 versus 4.0 ± 0.9 mU/L).

**Figure 2:**
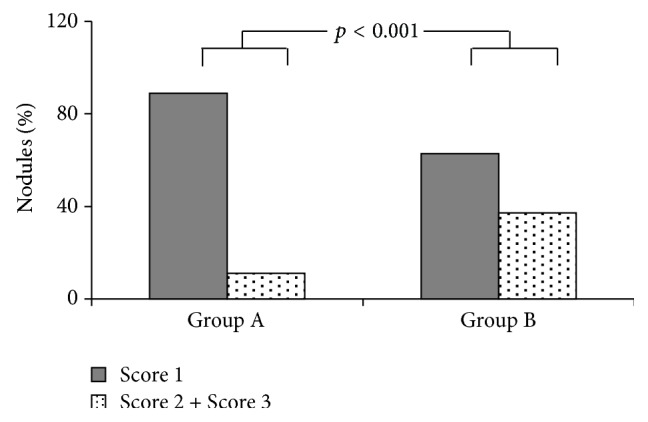
Elasticity score in nodules with benign diagnostic cytology in relation to thyroid hypoechogenicity pattern.

Following stratification, a significant different behavior emerged in the two groups (Figure 2). In detail, score 1 was found in 84.2% of benign nodules of patients with a mild-moderate ultrasound thyroid hypoechogenicity (sensitivity 75.0%; specificity 88.9%; positive predictive value 27.3%; negative predictive value 98.4%; accuracy 88.2%; *p* < 0.0001) and in 60% of patients with a marked thyroid hypoechogenicity (sensitivity 50.0%; specificity 62.8%; positive predictive value 5.9%; negative predictive value 96.2%; accuracy 62.2%; *p* = 0.715).

## 4. Discussion

As described by Gutekunst et al. thirty years ago HT is associated with a diffuse reduction in thyroid echo levels (hypoechogenicity) [19], with a correlation between serum TPOAb levels and the degree of hypoechogenicity [18, 20], that have been classified from mild-moderate to severe [17, 18]. After US introduction into clinical practice in the late nineteen sixties thyroid ultrasonography proved to be very effective in the diagnostic approach to thyroid disease [21].

Elastography has been proposed as a new and promising technique for thyroid nodules evaluation [8–10, 13]. The first evidence suggested that interobserver agreement is not reliable in the diagnosis of malignant lesions but recent data clearly shows a good interobserver and intraobserver reproducibility in detecting thyroid cancer [22, 23].

Magri et al. evidenced that shear wave elastography correctly defined the elasticity of thyroid nodules independently of the coexistence of autoimmune thyroiditis, being able to differentiate nodular tissue from the surrounding parenchyma [6]. The results of the present study confirm that elastography is effective in distinguishing between benign and malignant nodules in patients affected by HT, showing however a lack of accuracy in patients affected by HT presenting diffuse marked hypoechoic thyroid echo-structure (i.e., severe sonographic thyroid hypoechogenicity). On the contrary, two recent studies have shown that strain index reflects malignancy better than the elastoscore in HT patients [11, 12].

Taking into account the elastography performance in our patients without stratification by sonographic hypoechogenicity, our results also showed a lack of accuracy, but for the first time, we showed that elastography has a high performance when the thyroid echogenicity is not severe.

As already reported by Pedersen et al. [24] stronger inflammatory process has been shown in severe sonographic thyroid hypoechogenicity due to the diffuse infiltration of the thyroid parenchyma with lymphocytes and fibrosis [20, 25]. It is conceivable that a benign process, likely represented by nodular fibrosis, is responsible for the hard elastographic pattern of many thyroid nodules in patients with HT. In fact, histologically proven fibrotic nodules of the thyroid appear to be characterized by a high stiffness index at elastographic evaluation [26] and several hard nodules were detected in patients previously submitted to radioiodine treatment which is known to induce fibrosis [27]. Taking into account these studies, we have hypothesized that higher inflammatory process, lymphocytic infiltration, and fibrosis characterize HT with severe sonographic thyroid hypoechogenicity, but these reactions are also present in benign nodule(s) therefore reducing the accuracy of elastographic evaluation in these patients. Our results are in accordance with those of Scacchi et al. obtained in acromegalic patients, who showed that higher fibrosis developed in acromegalic thyroid patients justifying the worst performance of elastography in detecting cancer [28].

The present study has few limitations; firstly no histological data of benign nodules are given and secondly there is the relative small size of patients enrolled in the study. Another potential confounding factor may be the possibility that “benign nodules” were represented by pseudonodular fibrotic infiltration.

Unfortunately, we do not have histological data about cytologically proven benign nodule(s). Only a small number of patients (*n* = 26) with large benign nodules were submitted to thyroidectomy and in all these patients histology confirmed the benign nature of the lesions (data not shown). However, we must underline the accuracy in terms of sensitivity, specificity, and false negative and positive rate of FNAC [29, 30] to consider this procedure the gold-standard in differentiating benign from malignant thyroid nodules in many guidelines [5, 14]. Moreover, not only cytology [29, 30] but also elastography [13, 26] well distinguishes true nodules from pseudonodular ones. For these reasons we believe that the lack of histological data in our study cannot diminish the value of our results. Prospective large studies are needed to confirm our observation.

In conclusion our study has demonstrated a high prevalence of hard thyroid nodule at elastography evaluation in patients with HT. Interestingly this is particularly evident when the hypoechoic pattern of thyroid gland is severe, suggesting an extensive infiltration of the thyroid parenchyma with lymphocytes and fibrosis. The hypothesis that nodular fibrosis might account for this elastography pattern is conceivable but needs histopathological confirmation. Elastography evidences a lack of accuracy when it is performed in patients affected by HT presenting diffuse severe hypoechoic thyroid echo-structure.

## Figures and Tables

**Table 1:** The applied standard elastography color scoring system according to Rago criteria [13]. A score of 1 indicates even elasticity in the whole nodule, score 2 elasticity only at the peripheral part of the nodule, and score 3 no elasticity in the nodule and in the posterior shadowing.

Score	
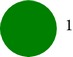	Elasticity in the whole nodule

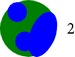	Intermediate elasticity in the nodule

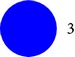	Low elasticity in the nodule

**Table 2:** Elasticity score of the 242 nodules submitted to cytology that resulted in benign nodules or carcinoma.

Elasticity score	Benign (*n*)	Cancer (*n*)	Tot. (*n*)
Score 1	182	2	184
Score 2	39	5	44
Score 3	9	5	14
